# KCNH2 mutation c.3099_3112del causes congenital long QT syndrome type 2 with gender differences

**DOI:** 10.1016/j.clinsp.2023.100285

**Published:** 2023-09-30

**Authors:** ZunPing Ke, Chao Li, Gang Bai, Li Tan, JunFeng Wang, Ming Zhou, JianHua Zhou, Shi-You Chen, Xiao Dong

**Affiliations:** a School of Public Health, Hubei University of Medicine, China; b Children's Medical Center, Taihe Hospital, Hubei University of Medicine, China; c Department of Ultrasonics, Taihe Hospital, Hubei University of Medicine, China; d Cardiovascular Center, Taihe Hospital, Hubei University of Medicine, China; e Department of Surgery, University of Missouri School of Medicine, Columbia, MO, USA

**Keywords:** LQTs, KCNH2, Kv11.1, Ventricular arrhythmias, Ventricular fibrillation

## Abstract

**Introduction:**

Long QT Syndrome (LQTS) is an inherited disease with an abnormal electrical conduction system in the heart that can cause sudden death as a result of QT prolongation. LQT2 is the second most common subtype of LQTS caused by loss of function mutations in the potassium voltage-gated channel subfamily H member 2 (KCNH2) gene. Although more than 900 mutations are associated with the LQTS, many of these mutations are not validated or characterized.

**Methods and results:**

Sequencing analyses of genomic DNA of a family with LQT2 identified a putative mutation. i.e., KCNH2(NM_000238.3): c.3099_3112del, in KCNH2 gene which appeared to be a definite pathogenic mutation. The family pedigree information showed a gender difference in clinical features and T-wave morphology between male and female patients. The female with mutation exhibited recurring ventricular arrhythmia and syncope, while two male carriers did not show any symptoms. In addition, T-wave in females was much flatter than in males. The female proband showed a positive reaction to the lidocaine test. Lidocaine injection almost completely blocked ventricular arrhythmia and shortened the QT interval by ≥30 ms. Treatment with propranolol, mexiletine, and implantation of cardioverter-defibrillators prevented the sustained ventricular tachycardia, ventricular fibrillation, and syncope, as assessed by a 3-year follow-up evaluation.

**Conclusions:**

A putative mutation c.3099_3112del in the KCNH2 gene causes LQT2 syndrome, and the pathogenic mutation mainly causes symptoms in female progeny.

## Highlights

• A mutation reported in this article appeared to be a definite pathogenic mutation of LQT2.
• This mutation exhibit gender differences in clinical symptoms and T-wave morphology.
• The female proband with this mutation showed a positive reaction to lidocaine attenuation test.

## Introduction

Long QT Syndrome (LQTS) is an inherited primary arrhythmia syndrome with a prevalence of 1 in every 2000 healthy live births.^1^ This autosomal dominant inherited disease causes palpitation, dizziness, syncope, and anoxic seizures secondary to ventricular arrhythmia, classically torsade de pointes with a risk of sudden death. There are 17 different subtypes of LQTS associated with monogenic mutations of 15 autosomal dominant genes.^2^ Mutations in these genes adversely affect cardiac ion channels, resulting in delayed repolarization of cardiomyocyte action potential and manifesting as prolonged QT intervals in the surface electrocardiogram. Based on the studies from Mayo's LQTS Clinic,^3^,^4^ there is substantial overlap in the distribution of QTc between healthy subjects and patients with genetically confirmed LQTS, which makes the diagnosis of LQTS challenging. Genetic testing of cardiac ion channelopathy mutations is now recommended as an essential component for diagnosis and familial cascade screening of LQTS patients.

LQT2 is the second most common subtype of LQTS, affecting approximately 30 % of congenital LQTS individuals. Genetic tests identify mutations of potassium voltage-gated channel subfamily H member 2 (KCNH2) involved in the reduction of IKr amplitudes which causes LQT2. Up to date, around 900 mutations in the KCNH2 gene have been reported in the Human Gene Mutation Database (HGMD). However, due to a paucity of gene- and variant-level evidence, only a small number of these mutations are found to be related to the clinical features. Ongoing efforts in the study of genotype, phenotype, mutation topology, and clinical therapies are important to reduce the risk of misinterpretation and diagnostic miscues.

In this study, we described the genotype, clinical, and ECG manifestations of a Chinese Han family with LQTS2. Targeted gene capture and next-generation sequencing were used to identify a deletion mutation, KCNH2(NM_000238.3): c.3099_3112del, in exon 13 of the KCNH2 gene. We further predicted three-dimensional structures of mutated protein using the homologous modeling method. Lidocaine attenuation testing was performed to investigate the therapeutic reaction to antiarrhythmic drugs. Cardiac events and ventricular tachycardia episodes were recorded with a three-year follow-up after dual chamber Implantable Cardioverter-Defibrillator (ICD) pacemaker implantation. Our clinical investigation of this pathological mutation provided new insight into the pathogenesis of LQTS and assisted the therapeutic decision.

## Patients and methods

### Ethics statement

This study was approved by the Medical Ethics Committee of Taihe Hospital, Hubei University of Medicine. All procedures were in accordance with the “Declaration of Helsinki” and the ethical standards of the Responsible Committee on human experimentation. Informed consent was obtained from all subjects or their legal guardians. The proband has provided informed consent for publication of the case.

### Proband and family investigation

The proband was a 31-year-old Chinese Han female, who was born at full term after a normal pregnancy and delivery. She was referred from a local hospital to Hubei University of Medicine affiliated Taihe Hospital because of recurrent ventricular tachycardia and ventricular fibrillation lasting for one day on June 6, 2017. She had experienced numerous palpitations, amaurosis, and syncope in the last 3-years, and the episodes usually occurred in the morning when there was a sudden loud noise during her sleep, and the episodes always appeared during her menstrual period. The symptoms recurred 2 weeks earlier and deteriorated for 2 days before she went to a local hospital. Potassium supplementation therapy was provided immediately in the local hospital, and defibrillation was given 3 times/day to treat the recurrent ventricular fibrillation.

**Fig. 1:**
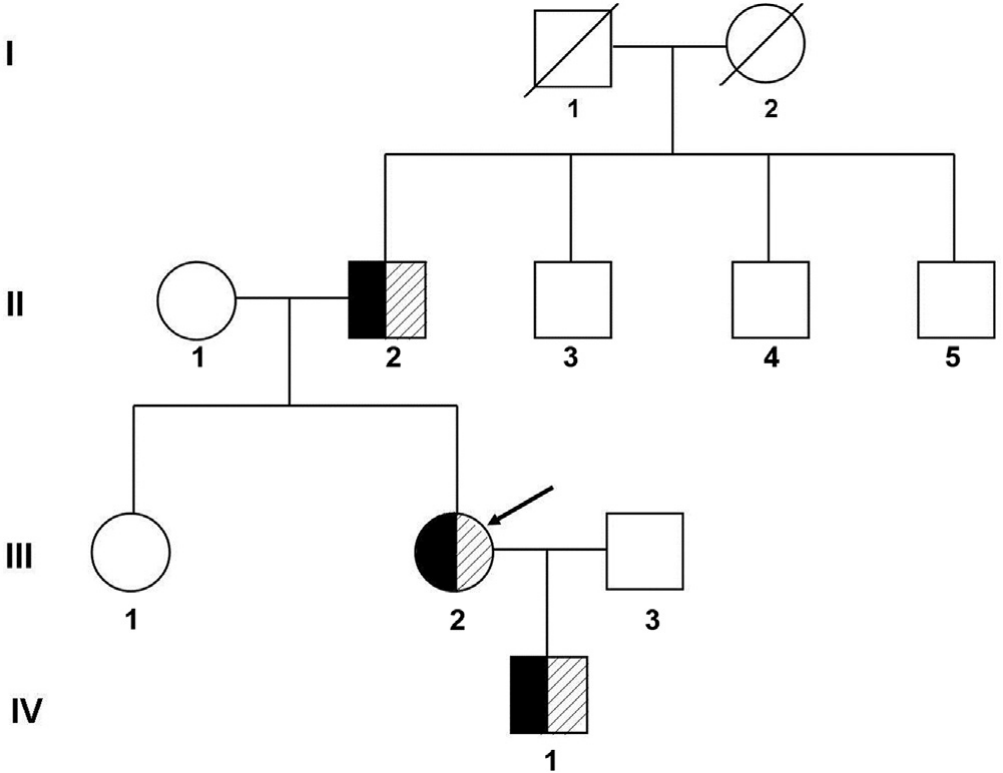
Pedigree of the family with long QT syndrome. I, II, III, and IV refer to the first, second, third, and fourth generations of the family, respectively. Black represents clinically diagnosed patients with Long QT syndrome, shadows represent carriers of pathogenic mutations, and the arrow indicates the proband.

After being transferred to our hospital, further laboratory tests and other examinations were performed, and electrocardiographic activities were monitored during medication. A family history investigation revealed that no similar symptoms were detected in other members. Eight individuals were enrolled for clinical and genetic studies and underwent a full physical examination, including QT interval assessment and T-wave morphology through ECG. The QT intervals were measured by the Tangent method and calculated by the Bazett correction formula, which gave the rate-corrected QT (QTc).^5^ The family members were clinically diagnosed as LQTS if they had a prolonged QT interval (QTc ≥ 470 ms for males; QTc ≥ 480 ms for females) or risk score ≥ 4 according to the Schwartz scoring scale.^6^,^7^ The family pedigree was shown in Fig. 1.

### Lidocaine attenuation testing

To investigate the patient's sensitivity to lidocaine, we performed a Lidocaine Attenuation Test (LAT) in a fasting state. Before intravenous infusion of lidocaine, the baseline QT, QTc, and RR intervals were obtained. Then, 2 mg/kg of lidocaine was infused with an intravenous line for 10 minutes followed by an infusion of 4 mg/min of lidocaine for 2 hours. QT intervals, RR intervals, and the derived Bazett's heart rate-corrected QTc values were recorded after the first 10-min infusion followed by recordings at 15-minute intervals for 2 hours. QT intervals were measured by the Tangent method as described above. All values were obtained from lead V5 as a mean of 3 consecutive beats. The LAT is considered positive if the QTc interval is shortened by ≥ 30 ms from baseline at any time point during the infusion according to Anderson's study.^8^

### Next-generation sequencing (NGS) and sanger sequencing

Next-generation sequencing was performed on the proband (III-8 in Fig. 1), her father, and her mother. Four milliliters of peripheral blood were collected and used to isolate genomic DNA (QIAamp DNA Mini kit, Qiagen GmbH). Target sequences were enriched using customized capture probes chips targeting 15 genes associated with LQTS, i.e., potassium voltage-gated channel subfamily Q member 1 (KCNQ1), potassium voltage-gated channel subfamily H member 2 (KCNH2), sodium voltage-gated channel alpha subunit 5 (SCN5A), Ankyrin 2 (ANK2), potassium voltage-gated channel subfamily E regulatory subunit 1 (KCNE1), potassium voltage-gated channel subfamily E regulatory subunit 2 (KCNE2), potassium inwardly-rectifying channel subfamily J member 2 (KCNJ2), calcium voltage-gated channel subunit alpha 1 C (CACNA1C), Caveolin 3 (CAV3), sodium voltage-gated channel beta subunit 4 (SCN4B), A-Kinase Anchoring Protein 9 (AKAP9), Syntrophin Alpha 1 (SNTA1), potassium inwardly rectifying channel subfamily J member 5 (KCNJ5), Calmodulin 1 (CALM1), and Calmodulin 2 (CALM2). Genomic DNA was fragmented on an E220 focused ultrasonicator (Covaris, Inc.), then captured by custom-designed DNA probes (Agilent Technologies, Inc.) and amplified by PCR. The final products were sequenced with 150-bp paired-end reads on the Illumina Nextseq 500 platform. All identified variants were annotated according to the guidelines published by the Human Genome Variation Society (HGVS, http://www.hgvs.org/content/guidelines).

Sanger sequencing was performed to confirm the potential pathogenic variants and to segregate them among family members. The specific PCR primers (Forward primer 5’-TCTACCCCGCTCACCCAG-3’, Reverse primer 5’-TCTCCCTCTACCAGACAACACC-3’) were used for the amplification of exon 13 in Kthe CNH2 gene based on the reference sequences of the human genome from GenBank in NCBI (NC_000007.14). Genomic DNA was first denatured at 94°C for 5 minutes, followed by 30 cycles of 98°C for 10 seconds, 60°C for 35 seconds, and 72°C for 60 seconds. The PCR products were extended at 72°C for 5 minutes. The products were gel-purified and sequenced using the forward and reverse primers. Automated sequencing was performed at both ends on an ABI 377 automatic sequencer. NGS and Sanger sequencing were completed by Sino Path Diagnosis (Beijing, China).leted by Sino Path Diagnosis (Beijing, China).

### ICD implantation

Under local anesthesia with lidocaine, implantation of the ICD system (Medtronic, D284DRG, PZM626455S) was undertaken using standard techniques. The ventricular lead (6944-65cm, TDC122150V) tip with defibrillation coil was positioned in the apex of the right ventricle, and the position was confirmed by a single shot of fluoroscopy. The right atrial lead (4574-53cm, BBE183554G) was placed in the right auricle whilst continued to check on the location of the ventricle lead by fluoroscopy to maintain it in the correct position (Supplemental Fig. 1). All the lead pacing/sensing parameters were recorded.

### Follow up

The patient was convalesced and discharged with a prescription of propranolol (10 mg three times a day) and mexiletine (150 mg three times a day). At the 3 months, 1 year, and 3 year follow-ups, pacemaker-recorded parameters (such as threshold, R wave amplitude and impedance) and events (such as ventricular fibrillation and heart beating stop) were examined. The ambulatory Holter monitoring, and echocardiography were performed and analyzed.

## Results

### Clinical abnormality of patients with LQTS

12-lead ECG of the proband performed in the local hospital demonstrated recurrent paroxysmal ventricular tachycardia, and torsade de pointes (Supplemental Fig. 2 A‒C). Electrolyte analyses showed that potassium and Magnesium were in the normal lower limit (K^+^, 3.68 mmoL/L; Mg^2+^, 0.77 mmoL/L) while NT-proBNP, blood glucose, coagulation function, blood cell count, CRP, arterial blood gas, thyroid function, CKMB, troponin, renal and liver function were all in normal ranges.

**Fig. 2:**
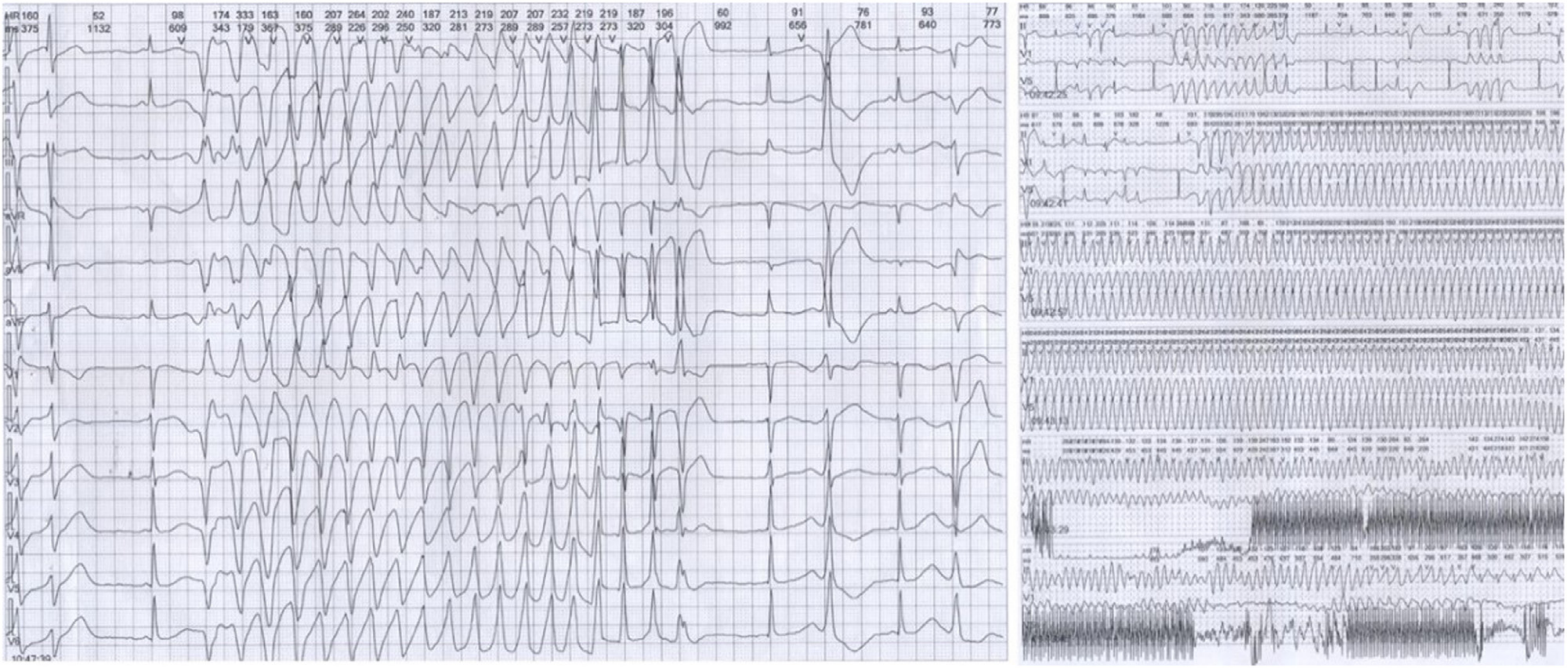
24h-ambulatory Holter monitoring recorded frequent ventricular premature beats, recurrent ventricular tachycardia, and fibrillation of the proband.

**Fig. 3:**
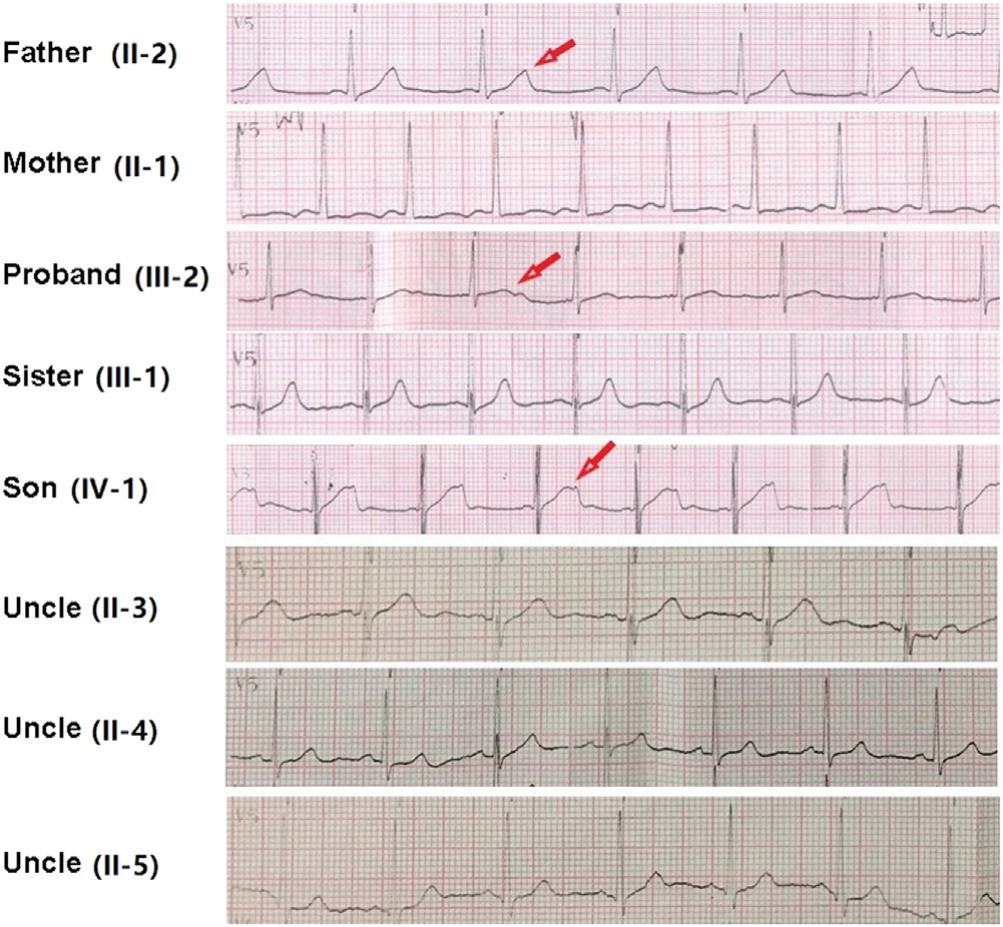
ECG of the proband and family members in V5 or V3 lead. Different T-wave morphologies were observed. Individuals with clinical diagnosed long QT2 syndrome including the proband (III-2), and her farther (II-2) and her son (IV-1) showed bifid or notched T-wave in ECG, pointed by red arrow.

12-lead ECG performed in Taihe Hospital showed a short PR interval of 110 ms and prolonged QTc interval of 523 ms (Supplemental Fig. 2D). Transthoracic echocardiography showed normal cardiac structure with the ejection fraction in normal lower limit (EF % = 53 %, Supplemental Fig. 3). 24h-ambulatory Holter monitoring recorded 8796 ventricular premature beats, 146 episodes paroxysmal ventricular tachycardias, and frequent torsade de pointes with some of them evolved to ventricular fibrillations (Fig. 2). The average heart rate in 24 hours was 68 beats/minute. The expression levels of antibodies of MPO, PR3, RF, dsDNA, nucleosome, SmD1, SSA, Scl70, Jo1 were all in the normal ranges (data not shown). Normal coronary artery was confirmed by enhanced cardiac Computed Tomography (CT) scan. Different T-wave morphology of the proband and family members were showed in Fig. 3, the female proband had a low amplitude and bifid T-wave, whereas 2 male mutation carriers had high amplitude and notch T-wave. Clinical characteristics of all available family members are summarized in Table 1. The female proband and her son showed short PR intervals (110 ms, 103 ms), whereas her father's PR interval was in normal range (123 ms). QT/QTc of the proband, her father, and her son were prolonged (410/482 ms, 461/461 ms, 450/483 ms). Schwartz's score was 8, 4, 5.5 for the proband, her father, and her son. According to the QTc and Schwartz score, the proband and her father and son were clinically diagnosed as LQTS, however, the father and son were asymptomatic.

**Table 1:** Clinical characteristics of family members in a Chinese Han family with LQTS. QT intervals were measured by Tangent method from lead II or lead V5 as a mean of 3 consecutive beats. QTc were calculated by Bazett correction formula.

**Subject**	**Sex**	**Age (y)**	**Heart rate (bpm)**	**PR interval (ms)**	**QRS duration (ms)**	**QT/QTs (ms)**	**ECG characteristics**	**Clinical symtoms**	**Schwart score**
Father, II-2	M	55	60	123	81	461/461	Prolonged QT subtle notched T-wave	No	4
Mother, II-1	F	54	101	190	70	330/434	Depressed ST segment flat T-wave	No	
Proband, III-2	F	31	81	110	66	410/482	Short PR interval prolonged QT bifid T-wave	Syncope	8
Sister, III-1	F	26	87	130	78	316/385	Normal ECG	No	
Son, IV-1	M	10	67	103	66	450/483	Short PR interval prolonged QT notched T-wave	No	5.5
Uncle, II-3	M	58	76	146	82	346/402	Normal ECG	No	
Uncle, II-4	M	65	81	136	65	349/401	Normal ECG	No	
Uncle, II-5	M	68	80	153	84	365/419	Normal ECG	No	

### Lidocaine normalized the QTc in proband with LQTS

Lidocaine tests showed QTc were reduced after lidocaine infusion in all time points. QTc interval shortened by ≥ 30 ms from baseline 482 ms to 450 ms after the initial dose and to 447 ms 45 minutes after the maintenance dose, suggesting a positive response to lidocaine treatment. Despite the QTc reductions in other time points being less than 30 ms, the attenuations were very close to the positive diagnostic criteria (Supplemental Fig. 4 and Table 1).

### Mutation detection and verification

**Fig. 4:**
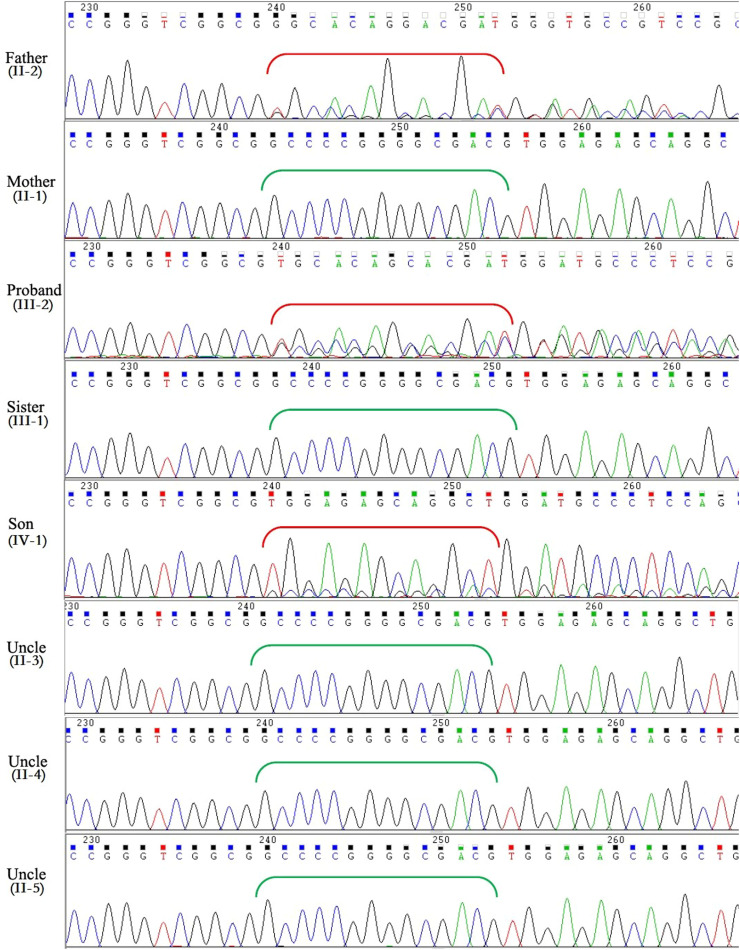
Sanger sequencing showed a heterozygous deletion-frameshift mutation, KCNH2(NM_000238.3):c.3099_3112del in KCNH2 gene in the proband, her father and her son, whereas other family members are normal. Red brackets indicate the frameshifted DNA sequence after deletion; green brackets show the original DNA sequence in individuals without mutation.

**Fig. 5:**
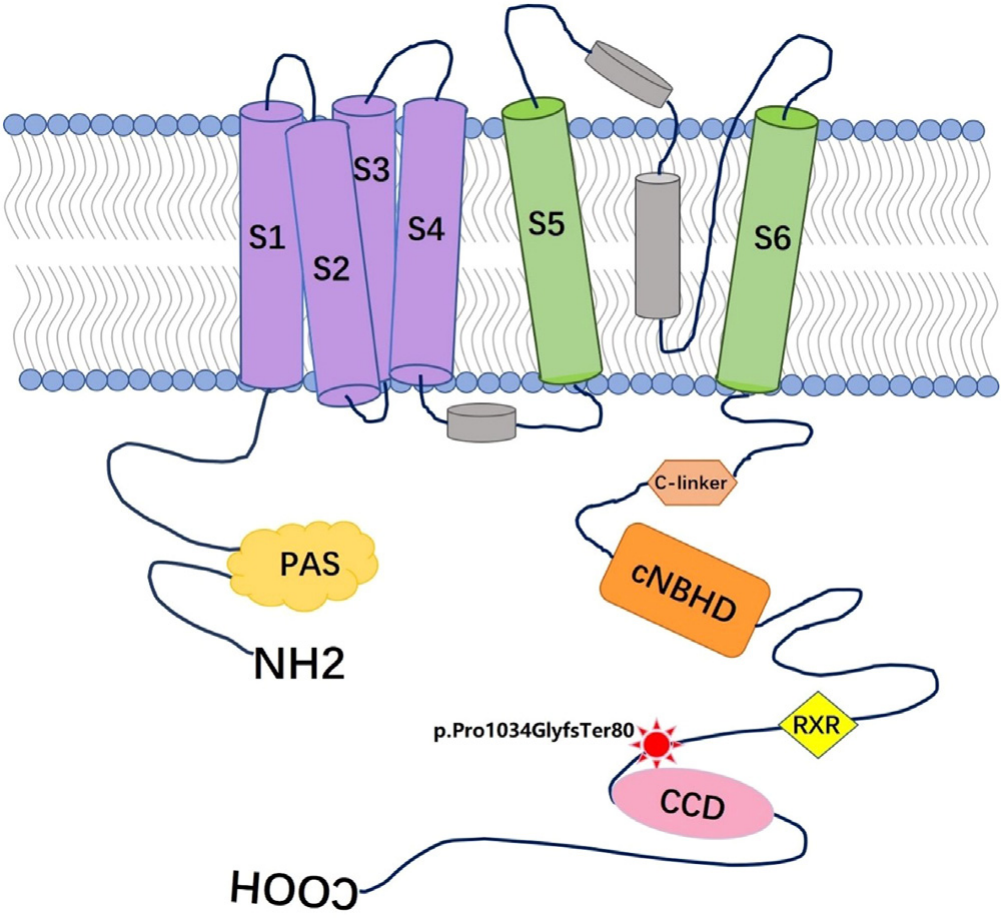
A structural model of the α-subunit of Kv11.1 protein and location of the mutation p.Pro1034GlyfsTer80 described in this family. Red star represents the mutation p.Pro1034GlyfsTer80, S1‒S6 is the transmembrane domains of Kv11.1; PAS is the Per-Arnt-Sim domain, (residues 26‒75); C-linker (residues 667‒744); CNBHD is the cyclic nucleotide-binding homology domain (residues 748‒865); RXR is the ER retention signal (residues 1005‒1007); CCD is the coiled-coil domain (residues 1036‒1074).

The variants with an allele frequency > 5 % in the dbSNP database, 1000 human genome dataset, Exome Aggregation Consortium (ExAC), and genome Aggregation Database (gnomAD) were excluded from the Next-generation sequencing data. By using the filtering criteria and analyzing the pipeline described in the Methods, a deletion-frameshift mutation, KCNH2(NM_000238.3): c.3099_3112del, in exon 13 of KCNH2 gene was discovered in both the proband and her father. The mutation was screened in genomic DNAs of all family members by Sanger sequencing. However, no mutation at this site was observed in other family members except the proband, her father and son (Fig. 4). This variant was first reported as a putative LQTS-associated mutation by Jamie in 2009,^9^ but nothing has been reported about relationship between the phenotype and genotype of this mutation. This heterozygous mutation resides in the distal C-terminus of the Kv11.1 channel, very close to the coiled-coil domain (Fig. 5), and causes a frameshift mutation after the amino acid 1033 (Arginine), which replaces the original 126 amino acids with 78 novel amino acids (p. Pro1034GlyfsTer80).

### Three years follow-up results

In the ICD implantation procedure, the right ventricle lead's pacing parameters were 0.5V (threshold), 10.8 mv (R wave amplitude) and 1000 Ω (impedance) while the right arial lead's pacing parameters were 0.5V (threshold), 3.1 mv (R wave amplitude) and 740 Ω (impedance). The initial pacemaker's programming control set the pacing rate at 80 beats per minute, and ATP function was turned on, 3 Burst, 3 Ramp, and alternate; defibrillation discharge energy was 35J when ventricular tachycardia rate > 180 beats per minute or ventricular fibrillation > 200 beats per minute. During the 3-month follow-up, the patient did not have any discomfort, and the pacemaker's programing control recorded 304 episodes of non-sustained ventricular tachycardia (> 4 beats, > 182 bpm), however, no discharge was recorded. After a 3-years follow-up, no cardiac event occurred, and the pacemaker's programming control recorded only 47 episodes of non-sustained ventricular tachycardia whilst no discharge was recorded since the first time follow-up. 12-leads electrocardiogram showed that QTc was 473 ms without pacing and 459 ms with pacing (Supplemental Fig. 5), echocardiography is normal and the ambulatory Holter monitoring showed no ventricular tachycardia were recorded in 24 hours.

## Discussion

15 autosomal dominant genes have been found to be associated with LQT1-15 subtypes, including KCNQ1, KCNH2, SCN5A, ANKB, KCNE1, KCNE2, KCNJ2, CACNA1C, CAV3, SCN4B, AKAP9, SNTA1, KCNJ5, CALM1, CALM2.^2^,^10^ Among these mutations, LQT1 (KCNQ1) and LQT2 (KCNH2) are the most common types of LQTS, accounting for more than 70  % of LQTS, whilst LQT3 (SCN5A) is responsible for less than 10 % of the cases. Clinically, cardiac events with LQT1 are usually triggered by exercise whilst a sudden startle or loud noise and emotional stress are potential triggers for LQT2, and LQT3 usually have events at sleep. On the surface electrocardiogram, LQT1 manifests as a broad-based and symmetrical T-wave with a prolonged QTc interval; LQT2 manifests as a bifid or notched T-wave that is asymmetrical and of low amplitude; LQT3 may manifest as a prolonged isoelectric interval preceding a relatively normal T-wave morphology. Although ECG and clinical features vary a great deal in various genotypes of the LQTS, not every case fits these characteristics, even the different members in the same pedigree have different T-wave morphologies. Some studies have shown that in genotype-positive LQTS families, the penetrance of pathogenic variants may be low for certain variants, indicating that the contribution of common variants to disease susceptibility may also contribute to variable disease penetrance.^11^,^12^ On the other side, the identification of a novel or an ambiguous variant of uncertain significance may rarely be clinically informative, which increases the risk of misinterpretation of genetic test results and further leads to potentially harmful diagnosis miscues. Along with the rapid development of genetic test technology, extensive clinical research of the common or uncertain variants is essential to the distinction of true LQTS-causative mutations from background genetic noise, finally, leading much more accurate diagnosis and better therapeutic approaches.

Among the 900 mutations in the KCNH2 gene, almost half of the suspected LQT2-causing mutations are missense mutations. Functional studies suggest that about 90 % of these mutations disrupt the intracellular transport or trafficking of the KCNH2-encoded Kv11.1 channel protein to the cell surface membrane.^13^ In this study, a Chinese Han family with a KCNH2 gene mutation is reported with the proband diagnosed as LQTS because of recurrent ventricular tachycardia and syncope with QTc of 482-523ms. Gene sequencing analyses and ECG examination of the whole family showed a deletion mutation KCNH2(NM_000238.3):c.3099_3112del for the proband, her father, and son. All of these three members exhibited prolonged QT intervals fit for the LQTS diagnosis criteria, whereas other family members without the mutation showed normal QT intervals. The proband's sister, who fortunately did not inherit this mutation from her father, showed normal QT interval without symptoms compared with the proband, indicating that this mutation may be a pathogenic mutation of KCNH2. This mutation was first reported by Jamie ^9^ in 2009 as a putative LQTS-associated variant by genetic test in 2500 cases cohort. However, there is a lack of detailed clinical manifestations described and functional investigations of this mutation conducted. The present report is the first to discover the genetic link of this mutation with LQTS in detailed clinical evidence.^14^ The mutation p.Pro1034GlyfsTer80 we reported is in exon 13 of the KCNH2 gene and the region of the distal COOH terminus (residues 1018–1122). Residues 1036‒1074 is a conserved coiled-coil domain named TTC or CCD, which is thought to be essential for subunit assembly/tetramerization.^15^ The mutation is just 3 amino acids preceding the TTC domain and causes a frame-shift translation of TTC, suggesting that amino acid changes in TTC region of Kv11.1 channel protein may cause QT prolongation and adverse clinical outcomes, supporting a novel functional role of TTC region in the normal physiology of KCNH2 channel.

Clinical data of the family show that this variant is not a putative mutation but a pathogenic mutation, especially for female individuals. Lidocaine attenuation testing of the proband with a positive response provides additional insight into the predictive value of this test. The patient was prescribed propranolol and mexiletine because beta blockers are regarded as first-line therapy for LQTS, but current standard therapies may not fully protect patients from occurrence of cardiac arrhythmias. ICD is another treatment proving effective in treating arrhythmia and preventing sudden death.^16^, ^17^, ^18^ After ICD implantation and follow-up for 3 years, non-sustained ventricular tachycardias are reduced from 304 episodes at the 3-months’ visit to 47 episodes at the third year's visit, and the QTc intervals are nearly normal (459 ms) with pacing at 80 beats/min. Moreover, the patient did not show any symptoms again during the follow-up. The present study along with others suggests that for patients with LQTS, therapeutic decisions should be made based on QTc, the presence of clinical and electrocardiographic features, and the confirmed pathogenic variant detected by genetic test.

Gender differences in congenital LQTS are well established in clinics, but the underlying causes remain to be determined. The follow-up study of a large congenital LQTS population of 1710 patients during a median of 71 years indicates that the female sex is an independent predictor of life-threatening events (HR = 1.70; 95 % CI 1.00 to 2.88; *p* = 0.048).^11^ In this,genotype-positive LQTS family, the seizure of clinical symptoms is also in a sex-specific predilection in women. The female proband suffered recurrent life-threatening ventricular arrhythmias arrhythmias and syncope, but the two male variant carriers did not show any symptoms and arrhythmia in daily life or routine health examinations, even though they also have prolonged QTc intervals and typical T-wave morphology in ECG, which could be due to the different androgen levels between the two genders. Interestingly, the morphology of T-wave differs slightly between males and females in this family, the female proband has a flat bifid T-wave in electrocardiogram which could lead to missed diagnosis, whereas the two male carriers have the typical LQT2 T-wave with clear notches. Publish data show that LQT2 males with pore location mutations have a significantly higher risk of cardiac events than non-pore location males (HR=6.01; 95 % CI 1.50–24.08; *p* = 0.011) whereas this association is not found in females.^19^ In the family of this study, the mutation is not in the pore formation location, which may explain why the two male carriers do not experience any arrhythmia attack although QTc intervals fit for the diagnosis criteria. As sex hormones have varying effects on the potassium currents in both genomic- and non-genomic-regulated pathways ^20^, further proteomics studies are needed to explain whether different mutations in Kv11 exhibit different reactions to estrogen.

## Conclusion

In this study, the authors confirmed a putative mutation KCNH2(NM_000238.3): c.3099_3112del as a potential pathogenic mutation. The clinical feature of this mutation is described for the first time. The family pedigree information revealed a gender difference with this mutation in clinical features and T-wave morphology. The female proband also showed a positive reaction to lidocaine attenuation test. The patient was treated by propranolol, mexiletine and ICD implantation, and no sustained ventricular tachycardia, ventricular fibrillation or syncope were observed in a 3-year follow-up monitoring.

## Authors’ contributions

ZunPing Ke responsible for data curtion and software, Chao Li and Gang Bai responsible for investigation, Li Tan, JunFeng Wang and Ming Zhou responsible for project administration, JianHua Zhou responsible for visualization, Xiao Dong responsible for writing original draft, funding acquisition and supervision, Shi-You Chen responsible for writing review & editing.

## IRB Information

The present study was approved by the Medical Ethics Committee of Shiyan Taihe Hospital. Reference number: 2019-34.

This observational study conforms to specific study guidelines of STROBE statement.

## Declaration of Competing Interest

The authors declare no conflicts of interest.

## Appendix Supplementary materials

Image, application 1
