# Supplementary material for: KCNH2 mutation c.3099_3112del causes congenital long QT syndrome type 2 with gender differences
Source: Clinics (Sao Paulo). 2023 Sep 30;78:100285. doi: 10.1016/j.clinsp.2023.100285 (PMC10562146; doi:10.1016/j.clinsp.2023.100285)
Supplement: Supplementary file 1 [file mmc1.docx]

**CLINICS-D-23-00138 - Supplementary Material**

**Table 1** Lidocaine attenuation testing shows QT intervals, RR intervals, and the derived Bazett’s heart rate corrected QTc values in different times after lidocaine infusion.

|  | **Time before lidocaine infusin** | **Time after loading dose** | **Time during maintenance dose** | | | | | | | |
| --- | --- | --- | --- | --- | --- | --- | --- | --- | --- | --- |
|  |  |  | **15 min** | **30 min** | **45 min** | **60 min** | **75 min** | **90 min** | **105 min** | **120 min** |
| QT (ms) | 410 | 378 | 381 | 391 | 398 | 399 | 387 | 397 | 363 | 386 |
| QTc (ms) | 482 | 450 | 459 | 454 | 447 | 463 | 455 | 467 | 453 | 459 |
| RR interval (ms) | 740 | 700 | 692 | 740 | 786 | 745 | 719 | 718 | 642 | 710 |

**Figure 1** Anteroposterior chest X-Ray after ICD implantation.


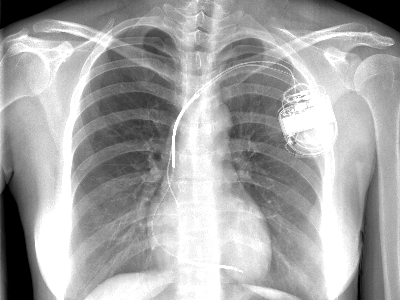


**Figure 2** ECG shows ventricular arrythmias and prolonged QT interval. A, B, C demonstrate recurrent paroxysmal ventricular tachycardia, torsade de pointes recorded in local hospital, D shows short PR interval (110 ms) and long QT intervals recorded in our hospital. (QT/QTc=461/523 ms, QT interval was measured by Tangent method from lead II and QTc was calculated by Bazett correction formula from lead II)

**Figure 3** Transthoracic echocardiography showed normal cardiac structure with ejection fraction in normal lower limit (EF% 53%).


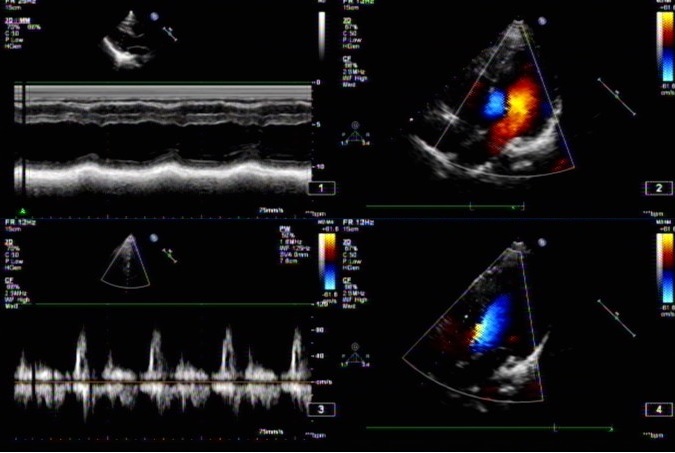


**Figure 4** Different time points of ECG in lidocaine attenuation testing. (A) Time before lidocaine infusion, (B) Time after loading dose infusion, (C) 15 minutes after maintenance dose infusion, (D) 30 minutes after maintenance dose infusion, (E) 45 minutes after maintenance dose was given, (F) 60 minutes after maintenance dose was given, (G) 75 minutes after maintenance dose was given, (H) 90 minutes after maintenance dose was given, (I) 105 minutes after maintenance dose was given, (J) 120 minutes after maintenance dose was given.


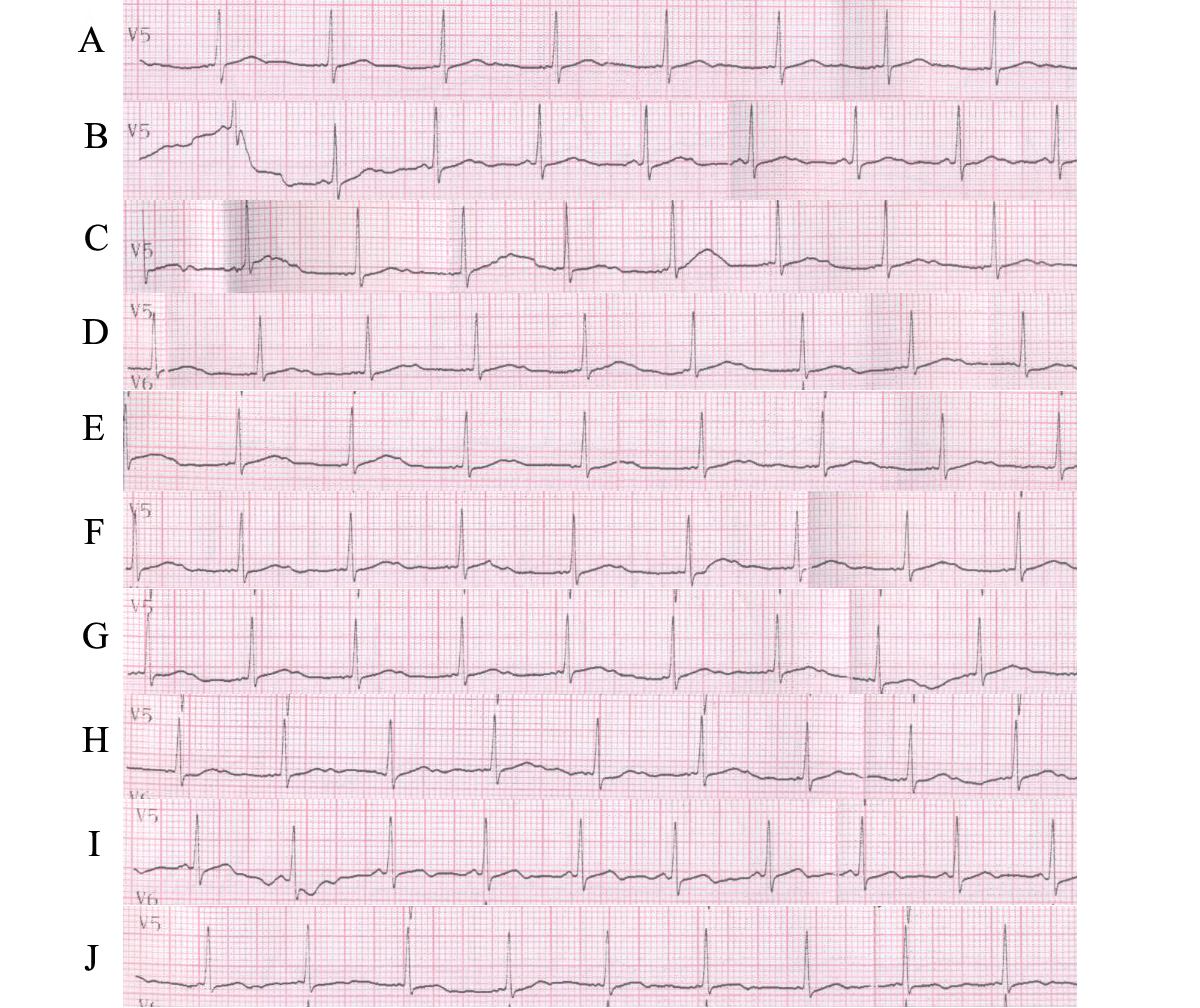


**Figure 5** ECG after 3 years follow-up. (A) ECG without pacing, (B) ECG with pacing
